# Arsenic-Induced Pancreatitis

**DOI:** 10.1155/2011/758947

**Published:** 2011-10-05

**Authors:** Sean Connelly, Krysia Zancosky, Katie Farah

**Affiliations:** Division of Gastroenterology, West Penn Allegheny Health System, 1307 Federal Street, Suite 301, Pittsburgh, PA 15212, USA

## Abstract

The introduction of all-*trans* retinoic acid (ATRA) and arsenic trioxide has brought about tremendous advancement in the treatment of acute promyelocytic myelogenous leukemia (APML). In most instances, the benefits of these treatments outweigh the risks associated with their respective safety profiles. Although acute pancreatitis is not commonly associated with arsenic toxicity, it should be considered as a possible side effect. We report a case of arsenic-induced pancreatitis in a patient with APML.

## 1. Introduction

Acute promyelocytic myelogenous leukemia (APML), first reported in 1957, accounts for approximately 10%–15% of cases of acute myeloid leukemia in adults [1–3]. Before the 1980s, this disease was among the most fatal at presentation or during induction; however, the introduction of all-*trans* retinoic acid (ATRA) and arsenic trioxide has brought about tremendous advancement in treatment strategies, with the majority of newly diagnosed and relapsed patients being able to be cured with these agents [4, 5]. Arsenic trioxide induces remission in 70%–85% of adults with newly diagnosed or refractory acute promyelocytic leukemia [6]. In general, the benefits of both therapies in patients with APML outweigh the risks associated with their respective safety profiles. However, we report a case of arsenic-induced pancreatitis in a patient with APML. 

## 2. Case Report

A 24-year-old female with a past medical history of cholecystectomy and APML presented to the hospital with a 2-day history of nausea, vomiting, and epigastric pain. Her oncologic history included induction chemotherapy with cytarabine, daunorubicin, and ATRA 1 year prior to presentation. One year after induction therapy, she had clinical relapse, and therapy was changed to arsenic trioxide at a dose of 0.15 mg/kg/day (17 mg daily), which was administered intravenously 5 days per week for an anticipated 20 dose total. On Day 11 of therapy, the patient developed the current symptoms of nausea, vomiting, and epigastric pain. Physical examination revealed tenderness in the epigastrium. With regard to laboratory data, the patient had an elevated amylase level (405 U/L; normal 30–110 U/L), an elevated aspartate aminotransferase level (43 U/L; normal 14–36 U/L), an elevated lipase level (4960 U/L; normal 6–75 U/L), and a low hemoglobin level (9.8 g/dL; normal 12.3–15.3 g/dL). Computed tomography (CT) scan of the abdomen/pelvis showed evidence of prior cholecystectomy. Right upper quadrant ultrasound revealed a normal pancreas, a common bile duct 3.5 mm without intrahepatic ductal dilation, a surgically absent gallbladder, and a normal liver. Magnetic resonance imaging (MRI) and magnetic resonance cholangiopancreatography (MRCP) revealed normal pancreatic morphology with no evidence of pancreas divisum. Bile aspirate was negative for microcrystalline disease. Triglycerides, antinuclear antibodies (ANA), and IgG4 levels were negative. Arsenic therapy was held during the hospitalization. The patient responded well to intravenous fluids, pain control, and antiemetics. Her biochemical parameters normalized by hospital day 8.

One week later, arsenic therapy was reintroduced at a dose of 0.1 mg/kg/day. On day 14 of therapy, the patient developed mental status changes, respiratory distress, hypotension, and recurrent pancreatitis with elevated amylase and lipase levels. CT of the abdomen and pelvis revealed heterogeneity of the pancreas consistent with pancreatitis. The arsenic trioxide was held, and succimer was initiated as a chelating agent for suspected arsenic toxicity. Blood and urine toxicology levels of arsenic were significantly elevated 25 days after cessation of therapy. Further workup for the altered mental status included EEGs which revealed generalized slowing consistent with toxic metabolic encephalopathy. The patient eventually required a tracheostomy and a percutaneous gastrojejunostomy and was transferred to a skilled long-term care facility.

## 3. Discussion

Arsenic is a naturally occurring element that is found in the earth's crust. It has a long been used in various commercial and industrial products, pharmaceuticals, and as an agent of deliberate poisoning [7]. Arsenic poisoning has been known to occur through industrial exposure from contaminated wine or moonshine but also through contamination of herbal preparations and nutritional supplements in addition to conditions involving attempted suicide. Arsenic is commonly also found in its organic and inorganic form in many foods such as dairy products, beef, poultry, rice, pork, and cereal. 

Although arsenic has long been known to act as a carcinogen in the human skin, lung, liver, kidney, and urinary bladder, arsenic trioxide (As_2_O_3_) has also been demonstrated to have anticancer activity in acute promyelocytic leukemia [8]. Several mechanisms of action may contribute to the anticancer activity of arsenic trioxide. Studies have found that in acute promyelocytic leukemia cells and cell lines, low arsenic trioxide concentrations (0.1 to 0.5 *μ*M) resulted in degradation of PML-RAR*α* proteins, relocation of PML into nuclear bodies, and partial differentiation. Higher concentrations (0.5 to 2 *μ*M) induced apoptosis through the formation of reactive oxygen species (ROS), including hydrogen peroxide and superoxide, which resulted in oxidative stress, DNA damage,   and the activation of Jun N-terminal kinase (JNK), triggering apoptosis [9–16]. It appears that there may be a role for direct islet cell injury of the pancreas, particularly endocrine cellular components of pancreatic islets. In addition, when examination of oral exposure of arsenic trioxide was performed in rabbits, elevated serum amylase activity and enhanced activity of oxidative stress have been found [17]. The presence of nitrite accumulation, lipid peroxidation, and resultant development of diabetes from chronic oral exposure of arsenic has also been described [17].

Arsenic has been suggested to be involved in cytotoxicity and genotoxicity by generation of nitric oxide and lipid peroxide [17]. Arsenic toxicity can result in a number of symptoms and side effects including cardiovascular, neur-ologic, dermatologic, hematologic, and hepatotoxic manifestations. Gastrointestinal side effects from arsenic poisoning are common and well described. Symptoms of acute arsenic toxicity include nausea, vomiting, and colicky abdominal pain. Profuse watery diarrhea is often seen. In chronic arsenic toxicity, neurological side effects and skin manifestations are especially common. 

The early clinical course of arsenic intoxication often mimics gastroenteritis. Symptoms can develop from 30 minutes to several hours following ingestion [18]. Values greater than 50–100 ug/24 hours are suggestive of arsenic intoxication [18]. A fatal dose ranges between 100 mg and 300 mg, although smaller doses may also be life-threatening [18, 19]. 

Arsenic-induced pancreatitis, whether secondary to intentional, accidental, or as part of chemotherapeutic therapy, is rare. An extensive review of the literature revealed 3 cases of arsenic-induced pancreatitis. In 1985, a case report describes a healthy 38-year-old female who presented with nausea, vomiting, and diarrhea 2 hours after ingesting herbal tea [18]. In this case, analysis of the patient's unused herbal tea bags as well as a variety of household items including kitchen powders, fertilizers, and garden supplies for arsenic were negative. Examination of family members revealed normal blood studies and no detectable arsenic by Gutzeit test in the urine. The patient was evaluated by a psychiatrist who noted the patient to be a reliable historian and without homicidal or suicidal tendencies [18]. Urinalysis for heavy metals showed an arsenic level of 9000 *μ*g/24 hours. The herbal tea was ultimately implicated as the source of arsenic. In 2006, there was a second case report of a 77-year-old male who suffered from acute pancreatitis during treatment of relapsed acute promyelocytic leukemia with arsenic trioxide after 25 days of therapy [20]. The third case involved a 26-year-old male with a history of surreptitious oral administration of a probable 10 g of arsenic trioxide who developed pancreatitis and toxic hepatitis. Despite chelation therapy with dimercaprol and dimercaptosuccinic acid, the patient died of multiple organ failure [21].

 Acute pancreatitis is not commonly associated with arsenic toxicity especially when used for chemotherapeutic purposes. Although seemingly rare, it should be considered as a possible side effect and should certainly be part of the differential diagnosis of drug-induced pancreatitis. It is unclear if dose reduction of arsenic trioxide aids in the prevention of subsequent episodes of pancreatitis. It is also unclear if therapy with arsenic trioxide should be discontinued after the first episode of pancreatitis provided all other etiologies are excluded. Further studies are needed to evaluate and analyze this clinical issue.
